# Response of *Cnidium officinale* Makino Plants to Heat Stress and Selection of Superior Clones Using Morphological and Molecular Analysis

**DOI:** 10.3390/plants11223119

**Published:** 2022-11-15

**Authors:** Hyung-Eun Kim, Jong-Eun Han, Hosakatte Niranjana Murthy, Hyuk-Joon Kwon, Gun-Myung Lee, So-Young Park

**Affiliations:** 1 Department of Horticultural Science, Chungbuk National University, Cheongju 28644, Republic of Korea; 2 Korea Disease Control and Prevention Agency, Osong-eup, Cheongju 28159, Republic of Korea; 3 Department of Botany, Karnatak University, Dharwad 580003, India; 4 Food Science R&D Center, Kolmar BNH Co., Seocho-gu, Seoul 30003, Republic of Korea

**Keywords:** catalase, clones, heat shock protein, heat stress, medicinal plant

## Abstract

*Cnidium officinale* is a medicinal plant cultivated for its rhizomes, which are used in Chinese, Japanese, and Korean traditional medicine. This medicinal crop is highly susceptible to heat stress and cannot be cultivated in regions of higher temperatures. In the present study, ten clones from Korea (clones 1, 2, 5, 6, 8, 11, 14, 15, 22, and 26) were evaluated for their heat tolerance in vitro at 25, 30, 32.5, and 35 °C, and growth characteristics including plant height, the number of leaves and roots were evaluated. The initial experiment was conducted to find the threshold level for significant damage to the plant, while the second experiment was to screen the germplasm to select heat-tolerant clones. Most of the clones were sensitive to heat stress (clones 1, 2, 8, 11, 14, 15, 22, and 26), and few clones (clones 5 and 6) could perform well at an elevated temperature of 32.5 °C. Molecular analysis of the expression of heat-responsive genes, including heat shock protein (*CoHSP*), catalase (*CoCAT*), and cystine protease (*CoCP*), was performed by quantitative real-time reverse transcriptase polymerase chain reaction (qRT-PCR) carried out with heat-tolerant and heat-sensitive clones. Two of the heat-tolerant clones (clones 5 and 6) showed significant expression of *CoHSP* and *CoCAT* genes at elevated temperature treatment. These clones can be used for further evaluation and cultivation.

## 1. Introduction

In China, Japan, and Korea, *Cnidium officinale* Makino (Family: Apiaceae) is a significant herbaceous medicinal plant that is widely cultivated for its rhizomes. The dried rhizomes are used in Chinese, Japanese, and Korean medicine as a tonic for the improvement of blood circulation, for overcoming problems with inflammation, and for the treatment of women’s menstrual problems [1]. Various pharmacological effects of rhizome extract have been documented, including antiangiogenic [2], anticancer [3,4,5], anti-diabetic [6], anti-inflammatory [7,8], immunomodulatory [9], analgesic [10], and antimicrobial [11,12] effects. Yang et al. [13] have shown that *Cnidium officinale* ethanolic rhizome extracts were responsible for the treatment of ischemic injury; as a result, rhizomes could be used as a recovery medication after plastic surgery, to treat peripheral blood circulation disturbances, and to treat postmenopausal issues. According to Kim [14] and Cha [15], *Cnidium officinale* extracts have beneficial effects in the inhibition of melanogenesis, reducing hyperpigmentation, and as a cosmetic component.

Numerous bioactive substances, including polysaccharides, polyacetylenes, and volatile alkylphthalide derivatives, have been found in the rhizomes of *Cnidium officinale* [9,16,17,18,19]. Polysaccharides such as β-1,6-linked D-glupyronosyl, β-1,4-linked L-arbinopyranosyl, and β-1,6-linked-galacopyronoslyl residues have been demonstrated to complement innate immunological activities [9,16,17]. Pregnenolone, coniferyl ferulate, vanillin, alkylpthalides including ligustilide, butyldenepthalide, senkyunolide-A, and senkyunolide B, C, D, E, F, G, H, I, and J were isolated by Kobayashi et al. [18], and these compounds were earlier reported to have antifungal and smooth muscle relaxing activities. Bae et al. [20] isolated falcarinodial (polyacetylene), 6-hydroxy-7-methody-dihydroligustilide, ligustilidiol, and senkyunolide H (alkylphthalides), which have demonstrated anti-inflammatory and anti-cancer activities. Mo et al. [21] isolated phthalide derivatives identified as senkyunolide B, 3-butylidene-4-methoxy-isobenzofuranone, 3-butylidene-6-hydroxy-isobenzofuranone, butylphthalide, 3-butyl-4-hydroxy-1(3H)-isobenzofuranone, senkyunolide F, neocnidilide, ligusilidiol, senkyunolide J, and Z-6-hydroxy-7-methoxy-dihydroligustilide, which possess pancreatic lipase activity and are beneficial for the regulation of obesity. Cnidilide (alkylphthalide), which was isolated by Tsukamoto et al. (2005) [22], is reported to have insecticidal activity, and this compound also possesses anti-inflammatory properties [23]. Ninomiya et al. (2016) [24] isolated several phthalides from *Cnidium officinale*, and senkyunolides G and H, in particular, showed significant triglyceride metabolism-promoting activity in high glucose-penetrated HepG2 cells, revealing the antidiabetic potential of these compounds. Luo et al. (2010) [25] demonstrated the neuroprotective effects of senkyunolide H.

High temperature or heat stress is reported to affect many aspects of plants such as growth, development, and productivity [26]. Furthermore, the effects of higher temperatures have been identified as a reduction in growth, alternation of photosynthesis, improper development, and alteration of secondary metabolism [27,28]. For example, in *Heracleum sosnowskyi*, exposure of plants to short-term heat stress (35 °C, 1 to 7 d) showed a decrease in chlorophyll and a lower rate of photosynthetic carbon dioxide assimilation. Additionally, the proline and anthocyanins increased in response to high temperatures [28].

The secondary metabolites produced during biotic and abiotic stress conditions enable the plants to counteract adverse effects. For instance, secondary compounds such as tocopherol and zeaxanthin, neoxanthin, and lutein act as antioxidants and may directly scavenge reactive oxygen species in response to photoinhibition [29,30]. Another contrasting example is *Arabidopsis thaliana*, in which an increase in the concentration of phenolics was reported during water stress [31]. Similarly, tolerant rice cultivars showed higher amounts of total phenolics and protocatechuic acid compared to sensitive cultivars for salinity stress [32]. Recent research advances support that products of secondary metabolism are important to alleviate the toxic effects of stress through interactions between membranes, proteins, and lipids which prevent membrane and protein disintegration [33] and through the expression of stress-responsive genes such as heat shock proteins and catalases. Heat shock proteins act as molecular chaperones that play a central role in plant heat tolerance by promoting the refolding of heat-denatured proteins and forming complexes [34]. Catalase exhibits antioxidant activity and removes the oxidative stress-inducing oxygen species [35].

*Cnidium officinale* is a crop that is highly sensitive to elevated temperatures [20,21,22,36,37,38]. Higher temperature affects the growth, photosynthesis, and yield of plants. Therefore, several shading technologies for the cultivation of plants have been developed by Nam et al. [36] and Kim et al. [37]. Polyploid plants have been synthesized with the objective of better heat resistance [39]; however, the yield and quality of rhizomes are drastically affected when these plants are cultivated at elevated temperatures. Finding the best genotypes that can withstand heat, drought, and salt stress and characterizing those genotypes by employing molecular, biochemical, and physiological factors involved in such resistance is one of the most popular techniques employed by plant scientists [40]. Such studies have been carried out in many medicinal crops, including fenugreek (*Trigonella foenum-graceum*) [41], yellow centaury (*Centurium maritimum*) [42], black cumin (*Nigella sativa*) [43], fennel (*Foeniculum vulgare*) [44], and cyclamen (*Cyclamen persicum*) [45]. However, such studies have not been carried out in *Cnidium officinale*. Therefore, in the current study, we assessed genotype/clonal variation for heat stress by using in vitro culture technique among ten clones of *Cnidium officinale* collected from different regions of Korea. The major objective was to identify the most heat-tolerant and heat-sensitive genotypes and to assess growth parameters. The initial experiment was conducted to find the threshold level for significant damage to the plants by exposing specific genotypes of *Cnidium officinale* to varying temperatures of 35, 30, 32.5, and 35 °C under in vitro cultures, and the second experiment was conducted to screen the germplasm at 32.5 °C to select the heat-tolerant genotypes. Several morphological characteristics were evaluated for screening the genotypes. In addition, the expression levels of heat stress-responsive genes such as heat-shock protein, catalase, and cysteine protease were used as molecular markers to determine the heat-sensitive and heat-tolerant genotypes/clones.

## 2. Results

### 2.1. Morphological Changes Due to Heat Stress

**Figure 1:**
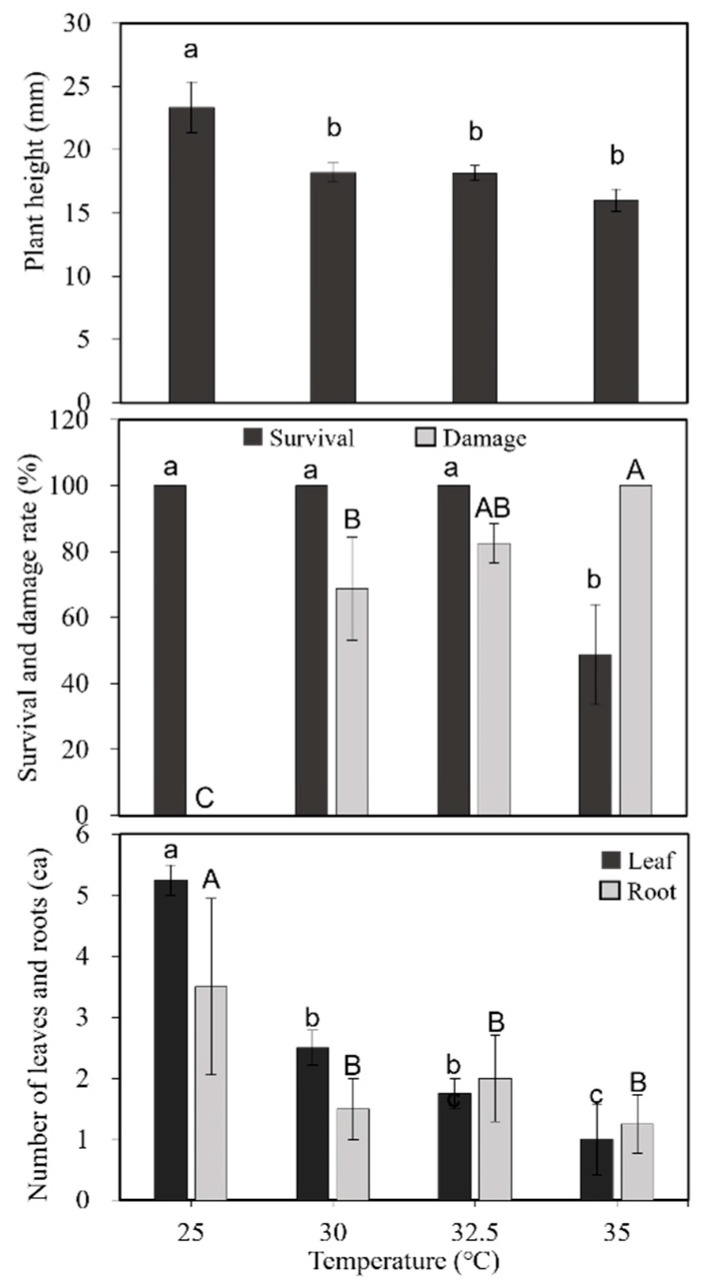
Effect of temperature treatments on plant height, survival, damage rate, number of leaves, and roots of *Cnidium officinale*—Clone 1. Different letters indicate mean values which are significantly different at *p* < 0.05 according to Duncan’s multiple range test (*n* = 10).

The initial experiment was conducted to check the temperature threshold level for significant damage to the *Cnidium officinale* plants, and data are presented in Figure 1. The height of the plants was 24 mm when they were grown at 25 °C, and the height of the plants gradually decreased with increments in temperature regimes of 30, 32.5, and 35 °C. The height of plants was 18 mm when plants were grown at 35 °C. The plant damage rate also increased with increments in temperature regimes, and all the plants showed yellowing of leaves and stem branches when plants were treated at 35 °C. The plant damage rate was 83% with the treatment of 32.5 °C (Figure 1). The plant survival rate showed a declining trend and 50% of plants survived with the 35 °C treatment; even though plants were alive, the majority of the portion stem and leaves had become yellowish and had a burnt appearance. The average number of leaves was five with the plants maintained at 25 °C, and a single leaf per plant was recorded with the plants with elevated temperature treatment (35 °C). Three to four roots were recorded with the plant growing at a temperature of 25 °C, and the number of single roots per plant was recorded with plants growing at 35 °C. These results depict that the threshold level for growing is 25 °C, and a temperature beyond 25 °C is detrimental to plant growth.

**Figure 2:**
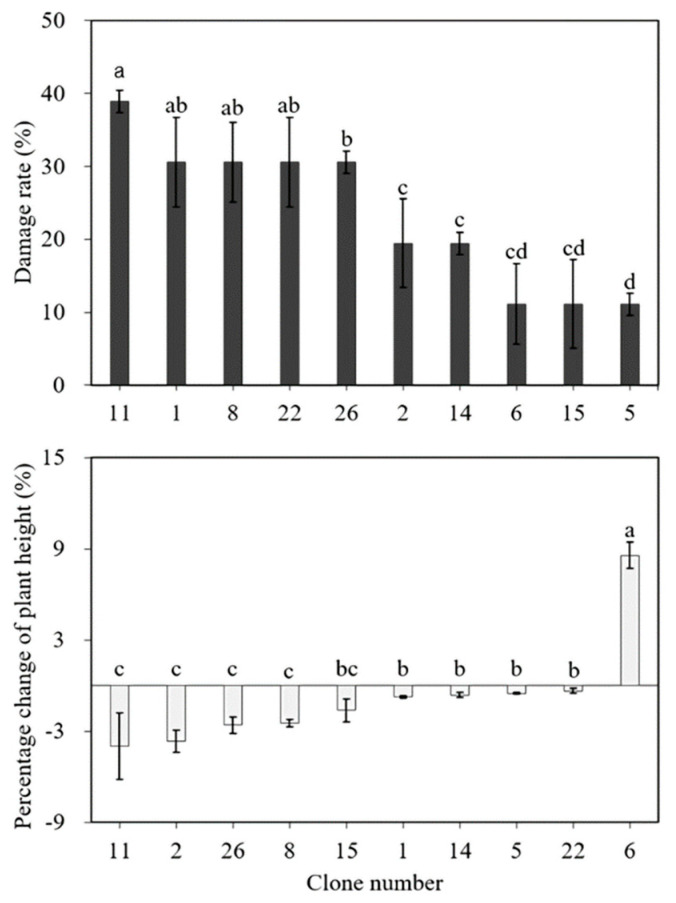
Effect of high-temperature treatment (32.5 °C) on plant height, and damage rate in various clones of *Cnidium officinale*. Different letters indicate mean values which are significantly different at *p* < 0.05 according to Duncan’s multiple range test (*n* = 10).

**Figure 3:**
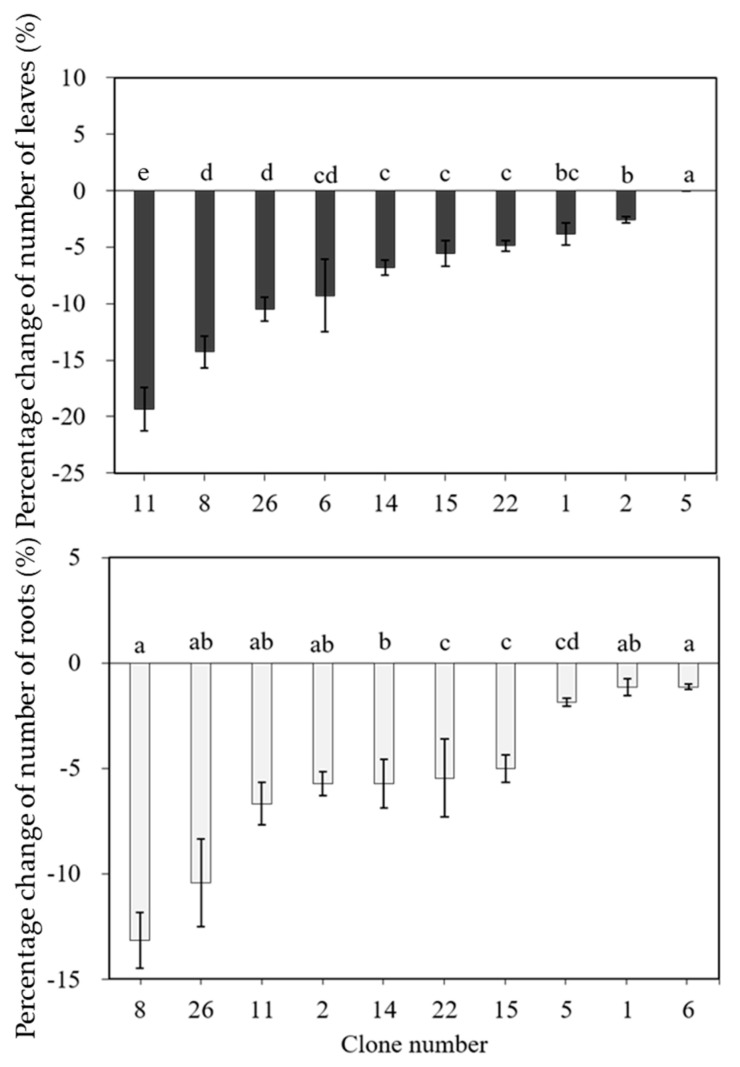
Effect of high-temperature treatment (32.5 °C) on the number of leaves and roots in various clones of *Cnidium officinale.* Different letters indicate mean values which are significantly different at *p* < 0.05 according to Duncan’s multiple range test (*n* = 10).

**Figure 4:**
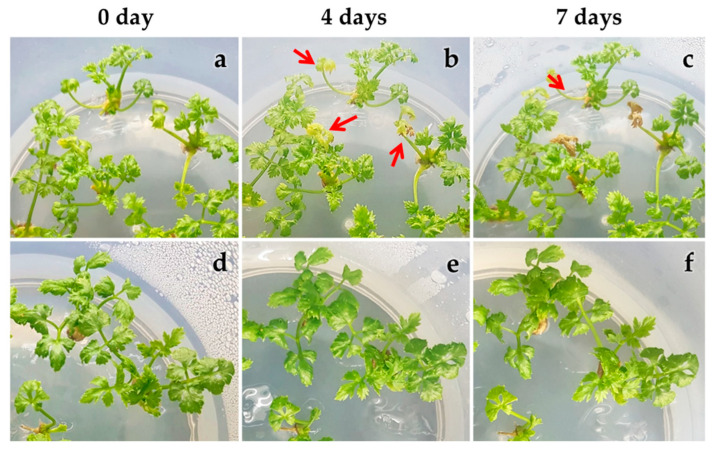
Effect of high-temperature treatment (32.5 °C) on the morphological changes of in vitro cultured plantlets: (**a**–**c**) heat-sensitive clone (clone 11; arrows indicate browning of leaves and stem branches); (**d**–**f**) heat-tolerant clone (clone 5). The red arrow denotes necrosis leaves and stems.

To assess the heat stress tolerance among varied clones/genotypes of *Cnidium officinale*, we conducted another set of experiments and cultured available clones of *Cnidium officinale*, i.e., clones 1, 2, 5, 6, 8, 11, 14, 15, 22, and 26, at 32.5 °C in vitro and assessed the growth parameters. The results are presented in Figure 2, Figure 3 and Figure 4. The damage rate was highest with clone 11, and 38% of plants showed symptoms of damage. The damage rate was also optimum for clones 1, 8, 22, 26, 2, and 14. However, the damage rate was less than 10% for clones 6, 15, and 5 (Figure 2). The percent of change in plant height was severely affected in all the clones except clone 6, and in clone 6, the average height of the plant was 8 mm (Figure 2). The percentage change in the number of leaves and the percentage change in the number of roots were optimum with all the clones (Figure 3). Figure 4 shows morphological changes with the in vitro cultured plants (clone 5 and clone 11) over 7 days. Clone number 11 had necrosis of leaves and branches which turned brown with the increment in culture duration (Figure 4a–c), whereas clone number 5, which is considered heat-tolerant, was healthy even though a few leaves turned yellow after 7 days in culture (Figure 4d,e). Based on the morphological analysis in response to heat stress, certain clones of *Cnidium officinale* were considered heat tolerant and certain clones as highly sensitive to heat stress: clones 5 and 6 were considered heat tolerant, and clones 8, 11, and 26 were considered highly sensitive to heat stress. These clones were used for the expression of heat-responsive genes.

### 2.2. Expression Levels of Heat Stress-Responsive Genes

**Figure 5:**
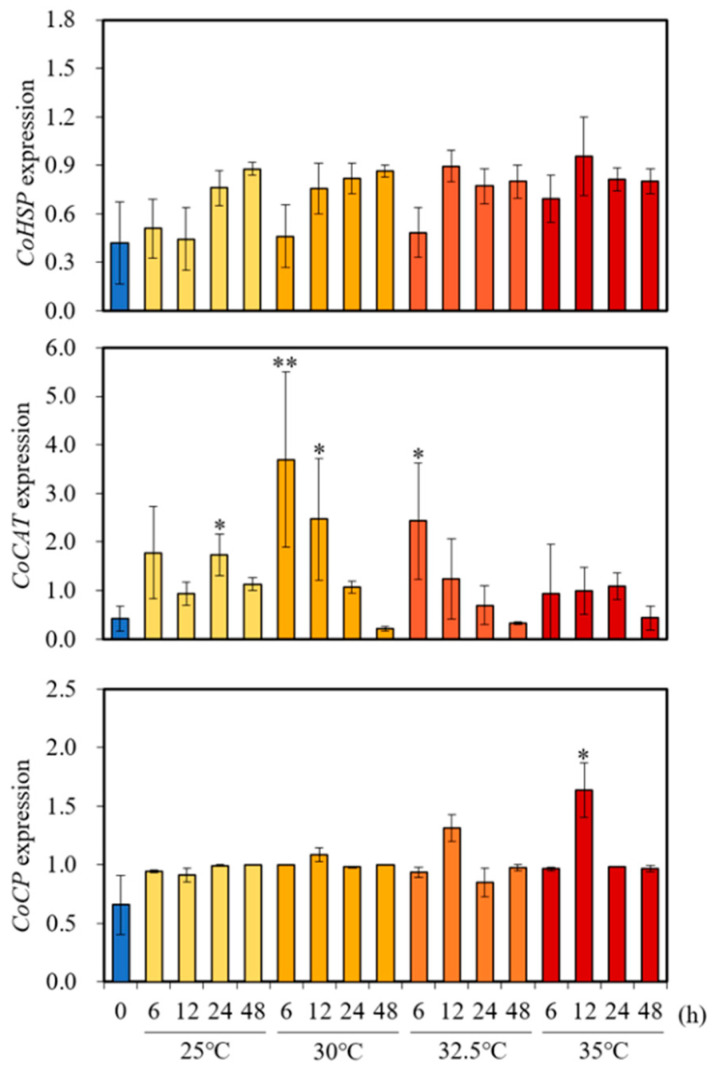
Effect of temperature treatments (25, 30, 32.5, and 35 °C) on the expression of heat stress-responsive genes (*CoHSP*, *CoCP*, and *CoCAT*) in *Cnidium officinale* (clone 1). *CoActin* gene was used as the reference gene. Asterisks represent significant differences in expression levels when compared to 0 h of treatment (*n* = 3, * *p* < 0.05, ** *p* < 0.01).

High-temperature stress severely damages plant growth and causes numerous morphological changes. Immediately after exposure to high temperatures, perception of signals and changes occur at the molecular level, altering the expression of genes and accumulation of transcripts, thereby leading to the synthesis of stress-related proteins as a stress tolerance strategy. In the current study, we performed q-RT-PCR to examine the expression levels of three heat-responsive genes, namely heat shock protein (*CoHSP*), catalase (*CoCAT*), and cysteine protease (*CoCP*), in *Cnidium officinale* plants exposed to different temperatures (25, 30, 32.5, and 35 °C) and at different intervals of time (0, 6, 12, 24, and 48 h). The results of the expression of *CoHSP*, *CoCAT*, and *CoCP* genes of clone 1 are presented in Figure 5. No significant difference was detected in *CoHSP* expression among the different treatments with different intervals of time. Unlike the above results, the *CoCAT* gene demonstrated significant expression levels at 25, 30, and 32.5 °C treatments with different exposure periods compared to zero h treatment/control. Similarly, the *CoCP* gene showed significant levels of expression with the plants exposed to 35 °C when compared to other treatments and the control.

**Figure 6:**
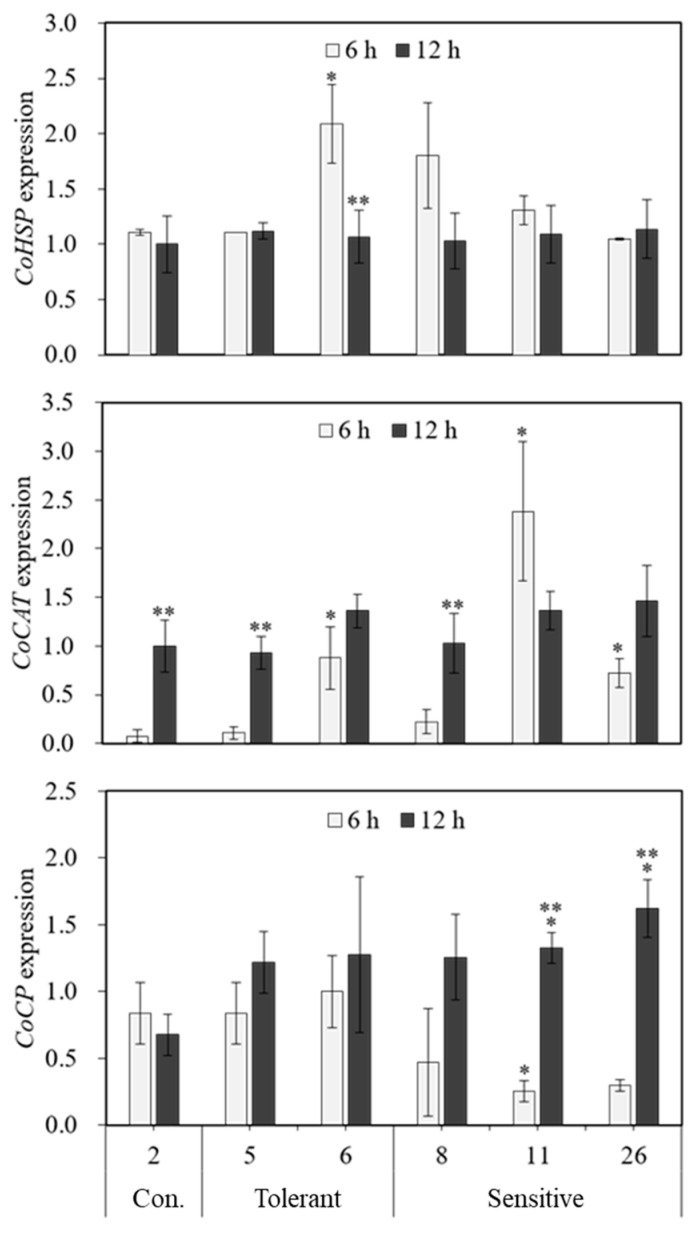
Effect of high-temperature treatment (32.5 °C) on the expression of heat stress-responsive genes (*CoHSP*, *CoCP*, and *CoCAT*) in *Cnidium officinale* heat-sensitive and heat-tolerant clones. *CoActin* gene was used as the reference gene. Asterisks represent significant differences at *p* < 0.05 (*n* = 3, * vs. clone 2, ** vs. 0 h).

The expression levels of *CoHSP*, *CoCAT*, and *CoCP* genes were also examined in two selected heat-tolerant clones (clones 5 and 6), heat-sensitive clones (clones 8, 11, and 26), and a clone that was moderately sensitive to the elevated temperature (clone 2), and the results were assessed at 6 and 12 h after heat treatment (32.5 °C) (Figure 6). The expression levels of *CoHSP* were highest in clone 6 and after 6 h when compared to clone 2/control. The level of expression was also the highest when compared to the 0 h treatment. Expression levels of the *CoCAT* gene were highest in clone 5 when compared to clone 2 after 12 h of treatment. *CoCAT* gene expression was also optimum in clone 6 after 6 h of treatment when compared to 0 h of treatment, and this gene was also expressed in heat-insensitive clones (Figure 6). The *CoCP* gene expression levels were significant in heat-sensitive clones rather than heat-insensitive/tolerant clones (Figure 6).

## 3. Discussion

*Cnidium officinale* is a temperate, highly heat-sensitive plant and it cannot be cultivated at temperatures beyond 28 °C, which affects photosynthesis, growth, development, and productivity [36,37,38,46]. The performance of the plant species under abiotic stress should be evaluated by assessing trait variability among the various genotypes to select the superior genotypes with higher stress tolerance [47]. The temperature induction response method is the most effective approach to evaluating the genotypes of *Cnidium officinale* for heat tolerance. The objective of the current study was to compare the relative responses of *Cnidium officinale* clones to heat stress at four different temperatures (25, 30, 32.5, and 35 °C) in an in vitro environment. Among varied temperature treatments, elevated temperatures (30, 32.5, and 35 °C) had a negative impact on the growth and plant height of *Cnidium officinale* plants in an in vitro experiment. The number of leaves and roots also decreased significantly. Noticeable necrosis and reduced growth of shoots and roots occurred at high temperatures. Similarly, heat stress has been reported to cause the inhibition of shoot and root growth, senescence, and abscission of leaves in *Pisum sativam* [48], *Oryza sativa* [49], and *Arachis hypogaea* [50]. We screened ten *Cnidium officinale* clones (1, 2, 5, 6, 8, 11, 14, 15, 22, and 26) for their ability to grow in vitro at high temperatures (32.5 °C). The majority of clones demonstrated necrosis and reduced growth characteristics. Clones 5 and 6 outperformed in all the growth characteristics tested, and these clones were selected as heat-tolerant clones for further molecular analysis. Similar to the current work, *Trigonella foenum-graceum* [41], *Centurium maritimum* [42], *Nigella sativa* [43], *Foeniculum vulgare* [44], and *Cyclamen persicum* [45] have all been analyzed for superior genotypes that can withstand heat stress.

Under heat stress, a series of physiological and biochemical reactions occur in plants, and normal protein synthesis is inhibited. At the same time, many new proteins are rapidly synthesized to maintain the physiological balance inside the cells. The most important of these are heat shock proteins. They are a class of conserved proteins that act as molecular chaperones and not only combine with the polypeptides being synthesized and assist them in folding correctly but also prevent the irreversible aggregation of denaturing proteins and maintain their biological functions against heat stress [51]. Varied pieces of evidence have shown that heat shock proteins are likely to be closely related to the recovery and survival of plants after heat stress [52]. Catalase is an important enzyme that plays a key role in the elimination of the toxic effects of oxidants and hydrogen peroxide and as an antioxidant [53]. One more valuable protein component which is accumulated in plants during heat stress is a protease, especially a cysteine protease, which acts as a key enzyme for the degradation of proteins required for the metabolic process [54]. The expression study of these genes in response to heat stress helps in the selection of superior genotypes in many crops. Therefore, in the current study, the expression levels of *CoHSP* (heat shock protein), *CoCAT* (catalase), and *CoCP* (cysteine protease) were analyzed by qRT-PCR. Initially, we tested clone 1 of *Cnidium officinale* at 25, 30, 32.5, and 35 °C and verified the expression of *CoHSP*, *CoCAT*, and *CoCP* genes at different intervals of time to assess the threshold level. The results revealed that catalase and cysteine protease genes have been expressed in different time intervals at elevated temperatures (30, 32.5, and 35 °C), and these results depict that *Cnidium officinale* is a heat-sensitive plant and cannot withstand temperatures above ambient temperatures (25 °C), as reported by other researchers [36,37,38,46].

In the second set of experiments, we compared the expression of *CoHSP*, *CoCAT*, and *CoCP* genes in selected *Cnidium officinale* heat-tolerant clones (clones 5, 6) and compared them with temperature-sensitive clones. The results of these experiments revealed that clones 5 and 6 showed significant expression of *CoHSP* and *CoCAT* genes at different intervals of time when compared to heat-sensitive clones. The *CoCP* gene was expressed in both heat-tolerant and heat-sensitive clones. Similar to the current results, the expression level of the heat shock gene was used to select heat stress-tolerant genotypes in *Arachis hypogea* (Kokanti et al., 2019) [47] and *Solanum lycopersicum* (Aldubai et al., 2022) [55]. Jha et al. (2021) [56] used gene expression profiling of catalase for the evaluation of salinity tolerance among pearl millet genotypes. In another study, Zhang et al. (2005) [57] verified the expression of the cysteine protease gene in *Festuca* species under heat stress conditions. The above results substantiate the usefulness of transcript profiling for heat-tolerant genes for screening tolerant genotypes in *Cnidium officinale*.

## 4. Materials and Methods

### 4.1. Plant Material

Ten clones of *Cnidium officinale* Makino available in the germplasm collection at the Department of Horticultural Science, Chungbuk National University, Korea, were used in the experiment. These germplasms were collected from different regions of Korea (Table 1). These clones were cultured in vitro on Murashige and Skoog (MS) medium [58] containing 0.1 mg·L^−1^ benzyladenine (BA) in a growth chamber/tissue culture room at 24 ± 1 °C under a 16 h light (35 µmol·m^−2^·s^−1^)/8 h dark photoperiod. Plants were maintained in vitro by subculturing once in 4 weeks.

### 4.2. High-Temperature Treatment and Analysis of Growth Parameters

In the initial experiment, *Cnidium officinale* clone 1 was used, and plants were subcultured on fresh medium and maintained in growth chambers at temperatures of 25, 30, 32.5, and 35 °C. Eight plants were cultured in each culture vessel and 10 replicates were maintained at each treatment for one week. Ten plants were randomly sampled 0, 5, 12, 24, and 48 h after treatments and cryopreserved for the analysis. One week after the culture treatment, the plants were assessed for plant height, the number of leaves and roots, damage rate, and survival rate. Growth parameters were calculated as follows: Survival rate = the number of survived plants/total number of plants inoculated × 100; Damage rate = the number of plants that turned brown/total number of plants inoculated × 100.

In the second set of experiments, plants of ten clones *Cnidium officinale*, viz., clone numbers 1, 2, 5, 6, 8, 11, 14, 15, 22, and 26, were cultured on Murashige and Skoog (MS) medium as described above, and maintained at temperature 32.5 °C. Ten plants were collected randomly, with each treatment at 0, 6, 12, 24, and 48 h cryopreserved for the analysis. Plants were assessed for their height, the number of leaves and roots, damage rate, and survival rate 7 days after treatment. Growth parameters were calculated as follows: Percentage of change in plant height = (final average height of plants-the initial average height of plants)/initial average height of plants × 100; Percentage of change in the number of leaves = (final average number of the leaves-the initial average number of leaves)/the initial average number of leaves × 100; Percentage of change in the number of roots = (final number of the roots-the initial-the initial average number of leaves)/the initial number of leaves × 100.

### 4.3. Gene Expression Analysis

#### 4.3.1. RNA Isolation and cDNA Synthesis

Frozen leaf samples (100 mg), with 10 replications, were pulverized in 1 mL of Nucleo-ZOL reagent (MACHEREY-NAGEL, Düren, Germany) with a stainless steel ball in TissueLyserII (QIAGEN, Hilden, Germany). Then, 400 µL of sterilized water was added to the tissue suspension and shaken vigorously for approximately 15 s. The sample was allowed to rest at room temperature for 15 min and then centrifuged at 12,000× *g* at 4 °C. The supernatant was transferred to a clean tube containing 1 mL of isopropanol. The mixture was allowed to rest for another 10 min and centrifuged at 12,000× *g* for 10 min at 4 °C. The supernatant was discarded, and the pellet was washed twice with 75% ethanol and then centrifuged at 3000× *g* for 3 min. The pellet was resuspended in 50 µL of sterile water by incubation at 65 °C for 5 min. Then, cDNA was synthesized from the isolated RNA using a Rever-Tra Ace^®^ qPCR Master Mix (TOYOBO, Osaka, Japan), and diluted to a concentration of 100 ng·µL^−1^ for gene expression analysis.

#### 4.3.2. Quantitative Real-Time PCR (qRT-PCR)

The expression of heat-responsive genes, including heat shock protein (*CoHSP*), catalase (*CoCAT*), and cystine protease (*CoCP*), was analyzed by qRT-PCR. First, 10 µL of SYBR Premix Ex Taq (Takara, Japan) and 2 µL of cDNA were mixed with 1 µL each of forward and reverse gene-specific primers. Then, 6 µL of sterile water was added to the sample to adjust the total volume to 20 µL. To obtain accurate results, air bubbles were removed from each sample. Then, qRT-PCR was performed on a CFX96 Touch™ Real-Time PCR Detection System (Bio-Rad, Hercules, CA, USA). The *CoActin* gene was used as an internal reference for data normalization. Primers used for qRT-PCR were designed based on the NCBI nucleotide database and are listed in Table 2.

### 4.4. Statistical Analysis

One-way analysis of variance (ANOVA) was performed to determine significant differences between each treatment and different clones. The statistical significance of the differences between mean values was then assessed by Duncan’s multiple range test at *p* < 0.05. All statistical analyses were performed using SAS 9.4 software (SAS Institute Inc., Cary, NC, USA).

## 5. Conclusions

In the present study, a total of ten genotypes/clones of *Cnidium officinale* were evaluated for heat tolerance, and two clones were suggested to be heat tolerant. Expression profiling of heat shock and catalase genes also demonstrated the heat tolerance of the selected clones. These contrasting genotypes of *Cnidium officinale* could be utilized for mining novel candidate genes imparting heat tolerance, aiming toward crop improvement. Further detailed study of these clones will facilitate an understanding of the molecular mechanisms of heat stress.

## Figures and Tables

**Table 1:** *Cnidium officinale* germplasm collected from different regions of Korea.

Clone Number	Place of Collection
Clone 1	Daegwallyeong, Republic of Korea
Clone 2	Daegwallyeong, Republic of Korea
Clone 5	Daegwallyeong, Republic of Korea
Clone 6	Daegwallyeong, Republic of Korea
Clone 8	Daegwallyeong, Republic of Korea
Clone 11	Bonghwa, Republic of Korea
Clone 14	Bonghwa, Republic of Korea
Clone 15	Bonghwa, Republic of Korea
Clone 22	Yengyang, Republic of Korea
Clone 26	Yengyang, Republic of Korea

**Table 2:** Primers for analysis of heat tolerance gene expression in *Cnidium officinale*.

Genes	Primer Sequence (5′ → 3′)	
*CoHSP* (Heat shock protein)	Forward	CGGAGAAGCAACGTGTTCGA
	Reverse	ACGCTGCAGTCTCTTTTCCG
*CoCP* (Cysteine protease)	Forward	CAGGTAGTTGCTGGGCATTT
	Reverse	TCAACGAGCTCTTGCTCAGA
*CoCAT* (Catalase)	Forward	TGTCTTCTTTGTGCGTGACG
	Reverse	CATAATGTGCCTTCCCAGCC
*CoActin* (Actin)	Forward	GGAAGCAGCAGGAATACACG
	Reverse	CGCTGTGATTTCCTTGCTCA

## Data Availability

Not applicable.
